# Adaptive Sports Bra Design for Adolescents: A Flexible Fit Solution

**DOI:** 10.3390/ma18174161

**Published:** 2025-09-04

**Authors:** Mei-Ying Kwan, Zejun Zhong, Kit-Lun Yick, Joanne Yip, Nga Wun Li, Annie Yu, Ka-Wai Lo

**Affiliations:** 1 School of Fashion and Textiles, The Hong Kong Polytechnic University, Hong Kong, China; 2 Faculty of Design, Architecture and Building, University of Technology Sydney, Broadway, Sydney, NSW 2007, Australia

**Keywords:** knitting, stretchability, sustainability, comfort

## Abstract

The development of adaptive and comfortable sports bras is essential for adolescents, who experience rapid changes in body morphology during growth. Traditional bras, often made with molded polyurethane bra pads, frequently fail to accommodate these variations, leading to discomfort and poor fit. This study investigates the design of a flexible-fit bra utilizing advanced knitting technology and bio-based materials, including organic cotton and renewable acetate, to enhance comfort and adaptability. The bra, crafted from bio-based yarns, offers stretchability, breathability, and fit, allowing it to adapt to various breast shapes and sizes. Such a bra design is particularly suitable for adolescents undergoing rapid growth. This study includes assessments of material properties and user feedback to evaluate the effectiveness of the design and identify areas for improvement. Positive results were reported from both material tests and subjective evaluations, confirming the effectiveness of the design. The seamless knitting minimizes irritation, while the inlay spacer fabric absorbs impact, and the pointelle structure improves moisture management. Adjustable components enhance adaptability and ensure a flexible fit. This study highlights the potential of knitted biomaterials for creating adaptive intimate apparel, offering a scalable solution for size-inclusive fashion.

## 1. Introduction

Growing awareness of the benefits of physical and mental health from participating in sports has led to a significant increase in women’s participation in active lifestyles. This trend is reflected in the booming global sports bra market, which was valued at US$43.7 billion in 2020 and is expected to reach US$93.7 billion by 2027 [1]. Demand for sports bras is particularly strong in the Asia-Pacific region, with 83.9% of Chinese women wearing a sports bra when exercising, compared to 67.2% in the United Kingdom [2].

Despite the surge in demand for sports bras, common issues such as poor fit and insufficient support remain, often leading to exercise-induced breast pain, friction injuries, and even long-term breast ptosis [3,4,5,6,7]. These issues are particularly severe for adolescent girls, whose growing bodies require flexible solutions to accommodate the rapid morphological changes of the breasts during puberty [8]. The current lack of research on adolescent bra design makes it difficult to improve the design.

Current sports bras fail to meet these needs. Traditional designs rely on rigid and pre-formed cups made from non-stretchable polyurethane foam that cannot adjust to individual breast shape or rapid growth [8]. Camisole tops that are traditionally worn by adolescents for exercise are usually loose-fitting, lacking proper breast support, protection, and fit, while the sizes are relatively vague. Additionally, over 91% of adolescent girls lack proper bra fitting education [9]. This challenge is even more acute with delicate breast tissue, asymmetric growth, and self-consciousness, which often prevents them from participating in physical activity. Worse still, 85% of adolescent girls fail to meet the World Health Organization recommended levels of physical activity, and breast-related discomfort is cited as a major barrier [10,11,12]. Adolescents who are active in sports have better life perception, and physical, mental, social, and emotional health [7,13].

To address this shortcoming, we propose an innovative seamless sports bra that utilizes advanced knitting technology and bio-based materials. Unlike traditional bras, our design uses inlay spacer fabrics to enhance stretchability, force absorption, and heat regulation. This research aims to advance the development of ergonomic bras for adolescents by evaluating bra performance through material testing and user feedback. Our designs prioritize fit, comfort, and sustainability, striving to empower young women to pursue the key benefits of size-inclusive fashion without compromise while pursuing an active lifestyle. Adolescent girls will be invited to take part in the subjective and objective bra evaluations via a wear trial. The overall fit and performance of the sports bra prototypes will be systematically evaluated and compared with commercial sports bras. Paramount to the design are the characteristics of the bra cup and breast support, as well as the design and material parameters of the sports bra, which will also be given.

## 2. Materials and Methods

### 2.1. Material Performance Evaluation

Studies highlighted that “comfort” and “fit” are the most critical factors in bra selection, with high breathability and thermal comfort being particularly favored [9]. Research confirmed that bio-based materials with a 60% organic cotton/40% renew acetate blend offer superior surface properties, which are crucial for comfort. These environmentally friendly materials enhance durability, breathability, and moisture wicking, while the addition of spandex-nylon blends improves tensile strength [14,15,16,17,18]. Therefore, 60% organic cotton/40% renew acetate blends and 15%/85% Spandex/Nylon will be used in this study as the surface yarn for bra production.

Prior to bra production, we fabricated and evaluated the performance of 11 different knit structures (Table 1). These structures were selected based on their potential to improve the thermal comfort, surface properties, and stretchability of sports bras. Each fabric underwent objective testing at a temperature of 20 ± 1 °C and a relative humidity of 65 ± 5% to select the optimal material for different bra components [16,19]. Standardized testing methods and specialized equipment were used to ensure accuracy and reliability.

Thermal comfort was assessed through air resistance, thermal conductivity, and water vapor transmission rate (WVT). Stretchability testing was also conducted to provide insights into the fabric’s elasticity, which is crucial for ensuring a flexible fit. By systematically analyzing these properties, this study aimed to develop a bra that surpasses conventional designs in terms of stretchability, breathability, and fit, while maintaining superior comfort.

### 2.2. Wear Trials

To understand the practical application of sports bras, this study employed a comprehensive approach to evaluate their performance through a systematic analysis of key design factors. The evaluation protocol consisted of laboratory testing (Phase One) and a four-week wear trial (Phase Two). Thirteen adolescent girls participated in this study and provided subjective feedback. Their demographics are shown in Table 2. The study design included a direct comparison of the experimental prototypes with a currently available commercial sports bra, enabling researchers to identify relative strengths of the new design and opportunities for improvement. This study adhered to strict ethical guidelines, with human subject ethics approval granted by the University Ethics Committee of Hong Kong Polytechnic University (HSEARS20230531003). All participants and their legal guardians provided informed written consent prior to involvement.

In Phase One, participants were required to run on a treadmill at a speed of 7 km/h for 8 min in a controlled laboratory environment, maintained at 70 ± 5% relative humidity and 28 ± 1 °C to simulate the daily use of the bras. Each participant was then given the most favorable sports bra for Phase Two evaluation. Phase II consisted of a four-week evaluation to assess the long-term comfort and practical usability of the bras under normal wear conditions. Participants wore prototype bras in their daily routines, providing insights into their actual performance. This longitudinal design also helps identify any comfort issues that may arise with repeated use.

User experiences were statistically analyzed using a 0–10 Likert scale, while open-ended questions provided detailed feedback on specific experiences. After the wear trials, researchers analyzed the collected data to improve the design. This evidence-based approach ensured that the research findings meaningfully contributed to the development of better-fitting and more comfortable sports bras for adolescent users.

### 2.3. Bra Designs

Since comfort is one of the most important qualities of sports bras [9], the sports bra prototypes strategically incorporate multiple structures in specific areas to optimize comfort and functionality. A suitable knit structure was selected after material evaluation for different bra components. The cup design should follow the natural curve of the breast and provide full coverage, with a stretchable, thick layer to eliminate the visibility of the nipple. The elastic inlay spacer structure previously investigated in [18] demonstrated excellent compression properties and shape conformity, effectively fit the breast shape, and protected the nipples while providing support and cushioning, and it maintained excellent stretchability compared to traditional laminated foam and spacer fabrics. This fabric is particularly suitable for bra cups, offering a balance between support, flexibility, and comfort [12,18,20,21]. The built-in design also prevents the cup from shifting during exercise.

The bra design incorporates different knit structures into a seamless adaptive sports bra. The bras are produced using a 14-gauge WHOLEGARMENT^®^ knitting machine (MACH2XS153-15L, Shima Seiki, Wakayama, Japan). This machine is widely used in seamless knitting due to its high precision and versatility, enabling it to produce complex knit patterns and structures. Five adaptive sports bra designs will be developed to provide superior breathability, comfort, and adaptability during physical activities like running and jogging.

A previous study comparing the properties of seven types of yarns found that a fabric featuring a blend of 60% organic cotton and 40% renew acetate enhances the breathability and eco-friendliness, and also exhibited lower friction and a smoother surface, making it suitable for the production of sports bras for adolescent girls, who are particularly sensitive to materials used in intimate apparel [18]. The built-in cups design features an innovative inlay structure that eliminates stitching, provides superior support and nipple protection, and maintains shape integrity during movement [18]. The seamless construction eliminates the friction-related discomfort common in traditional sewn bras. Adjustable components allow personalized fit adjustments, and the racerback design reduces strap slippage.

As shown in Table 3, Design 1 utilized pointelle 1 and a hollow design for increased breathability, while a hook-and-eye fastener provides adjustability. Design 2 changed the pointelle structure to further enhance breathability. Design 3 replaced the hook-and-eye with a hook-and-loop fastener and eliminated the hollow design, improving the aesthetics of the bra. Design 4 replaced the inlay structure on the upper part of the cup with pointelle stitches to increase breathability. Design 5 incorporated a zipper in the center front panel to facilitate donning and doffing. These prototypes address the functional needs of adolescent girls and improve the comfort of sports bras.

## 3. Results and Discussion

### 3.1. Material Tests

**Figure 1:**
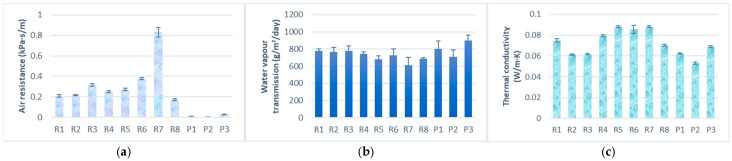
(**a**) Result of air permeability test; (**b**) result of water vapor permeability test; and (**c**) result of thermal conductivity test.

A total of eleven knit structures were developed and underwent objective evaluation. Air resistance is a critical factor in ensuring breathability, which directly impacts wearer comfort, especially during physical activities. The air resistance of different fabric structures was evaluated to determine their suitability for various parts of a sports bra. The test results, shown in Figure 1a, show that the pointelle structures offer the lowest air resistance, making them ideal for use in breathable zones such as the front and back panels. In contrast, the inlay structure (R7) exhibits poor breathability due to its three-layer sandwich-like structure, limiting its use in high-ventilation areas. However, the inlay structure provides excellent multi-directional support without compromising stretchability and has strong compression performance and load-bearing capacity, which can effectively provide support for the user, making the inlay structures suitable for making bra pads [18].

WVT measures a fabric’s ability to allow water vapor to pass through it, which is crucial for moisture management and maintaining dryness during physical activities. Figure 1b shows that pointelle sample P3 had the highest WVT, averaging 902 g/m^2^/day, followed by P1 (801 g/m^2^/day), while P2 achieves 712 g/m^2^/day. The excellent performance of the pointelle structure makes it an ideal choice for areas that require efficient moisture wicking, such as the back panel.

Figure 1c illustrates the variation in thermal conductivity among the tested samples. Thermal conductivity is a measure of a material’s ability to conduct heat, with lower values indicating better insulating properties. This property is crucial for maintaining thermal comfort. Results show that the inlay structure (R7) has the highest thermal conductivity, making it ideal for heat dissipation in areas such as bra cups.

### 3.2. Subjective Evaluations

In Phase One, the five prototype designs were compared against a commercial racerback sports bra. The subjective results, shown in Table 4, show that bra D1 scored high in fit (9.06) and softness (8.88), but low in support (7.56) and comfort (8.13). Bra D4, constructed mainly of single jersey, achieved an outstanding score in softness (9.50). However, evaluation of thermal comfort showed that the single jersey fabric was less breathable than the pointelle structure in bra D3 (Figure 1a). In contrast, bra D3 showed a more balanced performance, with high scores across key comfort metrics (ease of wear: 8.50, support: 8.38, comfort: 8.63, fit: 8.88, softness: 8.69), making it the most favorable choice among participants. Comfort and fit are particularly important for adolescents in their choice of sports bras [9]. The commercially available bra, however, received the lowest ratings, especially in satisfaction (7.00), ease of wear (7.09), and comfort (6.91). Participants ranked it as the least preferred choice due to itchiness from the materials of Polyamide and elastane, and a perceived lack of breathability. Furthermore, the bra lacked adjustment options to fit the body, which creates difficulties in donning and doffing. Hook-and-loop fasteners in the proposed bras may have had a positive impact on the wearing experience. When participants wore bras D1, D2, D4, and D5, which use hook-and-eye fasteners, they experienced fastening difficulties and itchiness around the fasteners.

In Phase Two, a four-week wear trial with 13 adolescents was conducted with bra D3. Participants were asked to provide information on the number of days they wore the bra and their exercise habits. Results showed that participants wore bras 3.3 to 4.3 days per week for activities such as running and volleyball. As shown in Table 5, the long-term evaluations of the D3 revealed high user ratings for ease of wear (8.6), fit (8.4), comfort (8.4), softness (8.5), and range of motion (8.5). This is likely due to adaptation to the sports bra. In addition, the hook-and-loop fastener and highly flexible fabric facilitated the ease of wear and fit, the materials and structures selected created a soft handfeel, and the racerback design did not hinder arm movement and prevented the shoulder straps from slipping, enhancing comfort and a sense of security.

The results show that overall satisfaction with the bras was generally high and stable, with many participants expressing appreciation for the design. The users were also satisfied with how well the bras conformed to their bodies. Most participants found the bras easy to wear, noting that they could slip them on without much effort. Range of motion was generally good, with scores ranging from 8.2 to 8.7, indicating that users felt unrestricted in their movements. The degree of itching (3.5) and pain (2.1) remained low. Comfort ratings varied slightly but stayed high, suggesting that the design provided good comfort in continuous wear.

For adolescents, comfort is a holistic blend of the physical and psychological. It was often mentioned by the subjects after the trial that they felt comfortable because the bra provided ample support while blending seamlessly under uniforms and T-shirts without visible straps or lines. This provided them with a sense of psychological security, allowing them to fully engage in exercise without fear.

**Figure 2:**
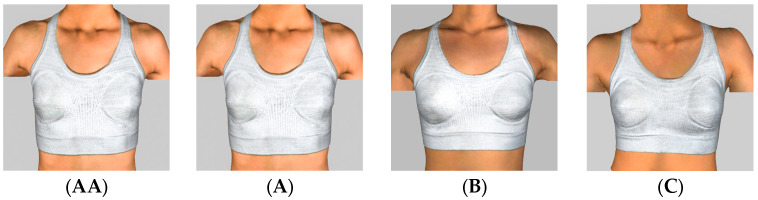
Subjects with cup sizes **AA**–**C** wearing Bra D3.

Figure 2 shows images of subjects wearing bra D3 with cup sizes ranging from AA to C. The results show that the designed bra provides a good fit across various cup sizes, effectively demonstrating a flexible fit design. Appropriate coverage, especially in the side areas, can enhance breast support and stability. The full-cup bra design can also effectively reduce breast movement by increasing coverage [22,23].

### 3.3. Bra Design Parameters and Performance

**Figure 3:**
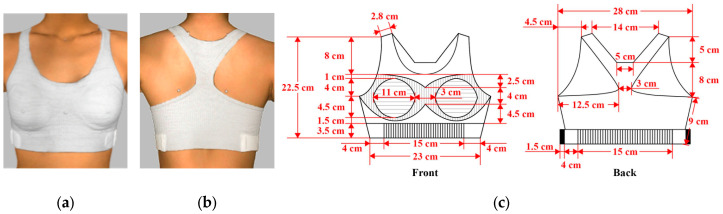
Scanned image of bra D3: (**a**) Front; (**b**) back; and (**c**) dimension configurations of the bra.

**Figure 4:**
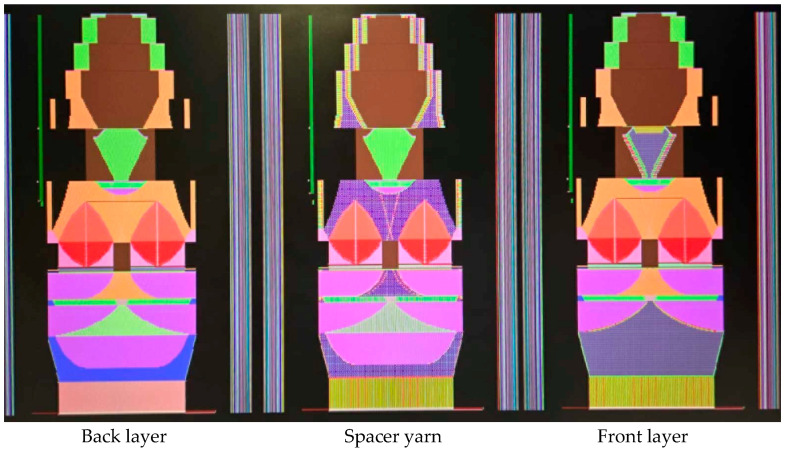
Bra knitted structure design.

After the laboratory wear trial, bra D3 (Figure 3 and Figure 4) was selected as the most favorable design. It uses a different knitting structure to enhance its stretchability and adaptability. The under band uses rib structure, which has extensive shrinkage in width and dimensional stability [2]. To provide better adjustability, Lycra was added to the under band to effectively increase the stretchability for ease of wear. The position of the hook-and-loop was moved to the side to facilitate the adjustment of the tightness of the under band and improve the aesthetics of the bra. The literature shows that the comfortable pressure range on the breast is between 0.96 and 1.355 kPa [24]. Some adolescent girls have a low tolerance to bra pressure when wearing a bra for the first time. Therefore, the pressure of the under band can be controlled with adjustable features to achieve optimal pressure.

The front and back panels use pointelle structure for high breathability, making it easier to evaporate sweat [25]. Racerback design was used in Bra D3. Previous studies have found that it can prevent the shoulder straps from slipping off the shoulders without reducing the overall breast support [25,26]. This design can reduce shoulder pressure by shifting the weight of the breasts to the center of the body [26]. In addition, the bra body and cups are connected by a stretchable knit structure, which provides enough space for breast enlargement. The knitting parameters of bra D3 are presented in Table 6. Knitting parameters, such as stitch density, determine the fabric’s flexibility and support. For instance, a tighter stitch enhances support, while a looser structure improves flexibility. These adjustments ensure the bra meets the dynamic needs of adolescent girls during exercise, directly affect the functionality of the bra, and are crucial for creating a sports bra that meets the specific needs of adolescent girls.

The performance of materials used in bra D3 is summarized in Table 7. Based on WVT measurements, the under band has the highest permeability (780.77 g/m^2^/day), followed by the main fabric (712.40 g/m^2^/day) and the cups (556.20 g/m^2^/day). This unit, expressed in grams of water vapor passing through one square meter of material per day, directly reflects permeability, where a higher value denotes better moisture management.

In terms of thermal conductivity, the under band (0.08 W/m·K) demonstrated the most efficient heat transfer among all the evaluated bra components. Air resistance testing revealed that the main fabric permitted the greatest amount of ventilation. This high ventilation capability is essential for thermal regulation, as it allows heat and moisture to dissipate efficiently from the skin surface, minimizing perspiration and preserving a dry, comfortable microclimate during wear.

Surface performance showed that the bra cups had the smoothest surface (SMD: 0.65), which improved comfort against the skin, while the main fabric (SMD: 7.33) and under band (SMD: 5.55) were intentionally rougher to minimize slippage during movement. The organic cotton of surface yarn helps to create a uniform surface for desirable handfeel and comfort perception [18].

The Young’s modulus and tensile strength of various bra components were tested using the Instron 4411 Tensile Tester in accordance with EN 14704 and ASTM D575 standards, respectively. Tensile strength measures a material’s resistance to breaking under tension and was evaluated by stretching the materials to 50% of their original length. Material stiffness was measured by Young’s modulus, with a higher value indicating greater rigidity and resistance to deformation. The main fabric has a low Young’s modulus, meaning it is flexible and readily deforms under stress. In contrast, the bra cups have a higher modulus, indicating they are much stiffer and retain their shape under load. They can withstand significantly more force before permanent deformation occurs. The under band provides structural support and moderate flexibility.

These findings advance the design of sports bras for adolescents by demonstrating how knit structures can be engineered to address physiological needs and offer a seamless alternative that better accommodates breast growth. These results have important implications for both athletic performance and long-term breast health.

## 4. Conclusions

This study investigated the key factors influencing sports bra design for adolescent girls and evaluated their performance through both subjective and objective assessments. The proposed adaptive seamless sports bra offers excellent breathability and is fully adjustable to fit the changes of the body, while ensuring comfort and functionality, making it an ideal choice for young athletes. The inlay spacer fabric provides excellent support and durability, while the pointelle structures enhance breathability and moisture management. Future research could further refine the design by exploring adaptive materials for dynamic fit adjustments during exercise. Future studies will recruit more participants to quantitatively evaluate the effects of different bra designs on anatomical characteristics of asymmetric torso morphology and breast support from the perspective of breast biomechanics, in order to develop more precise design guidelines. Overall, the results indicated a positive experience with the bra. Participants particularly appreciated the design’s comfort and adaptability. These insights pave the way for developing sports bras that better meet the evolving needs of adolescents, promoting their long-term participation in active lifestyles.

## 5. Patents

A patent titled “An Inlaid Seamless One-size-fits-more Sports Bra Design” was filed on 5th November 2024 (Filed No. 202411218992.5) by the China National Intellectual Property Administration.

## Figures and Tables

**Table 1:** The eleven tested knitted samples.

Fabric Structure	Code	Microscopic (Technical Face)	Fabric Structure	Code	Microscopic (Technical Face)
1 × 1 Rib	R1	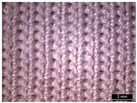	Inlay	R7	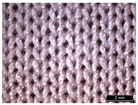
2 × 2 Links	R2	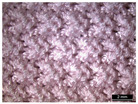	Tubular	R8	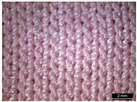
1 × 1 Links	R3	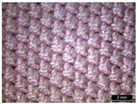	Pointelle 1	P1	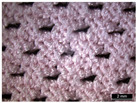
Single Jersey	R4	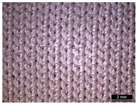	Pointelle 2	P2	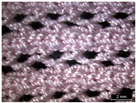
2 × 2 Purl	R5	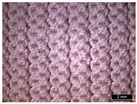	Pointelle 3	P3	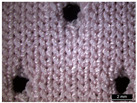
1 × 1 Purl	R6	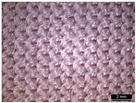			

**Table 2:** Demographics of participants (*n* = 13).

Parameters	Subjects’ Profile
Age (years)	12.85 (±1.35)
Height (cm)	153.52 (±6.52)
Weight (kg)	45.23 (±5.34)
Underbust (cm)	66.63 (±3.37)
Fullbust (cm)	74.50 (±5.33)

**Table 3:** A summary of sports bra designs.

Bra Design		Cups	Front	Back	Fasteners
1	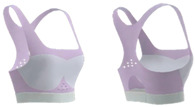	Inlay (R7)	Single jersey (R4), pointelle 3 (P3)	Racerback, hollow design, single jersey (R4), pointelle 3 (P3)	Hook-and-eye
2	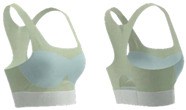	Inlay (R7)	Pointelle 1 (P1)	Racerback, hollow design, pointelle 1 (P1)	Hook-and-eye
3	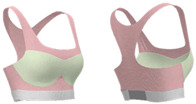	Inlay (R7)	Pointelle 2 (P2)	Racerback, pointelle 2 (P2)	Hook-and-loop
4	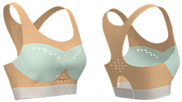	3/4 Inlay (R7)	Single jersey (R4), pointelle 3 (P3)	Racerback, hollow design, single jersey (R4), pointelle 3 (P3)	Hook-and-eye
5	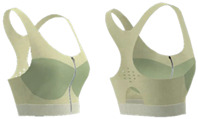	Inlay (R7)	Single jersey (R4)	Racerback, hollow design, single jersey (R4), pointelle 3 (P3)	Hook-and-eye, zipper

**Table 4:** Subjective evaluation of the laboratory wear trial.

Conditions	D1	D2	D3	D4	D5	B
Satisfaction	8.06	8.13	7.69	7.88	7.94	7.00
Ease of wear	8.69	8.25	8.50	8.13	7.81	7.09
No pain	9.19	9.13	8.94	9.13	9.31	9.18
Supportive	7.56	8.44	8.38	8.25	7.75	7.45
Comfort	8.13	8.63	8.63	8.63	8.19	6.91
Fit	9.06	8.69	8.88	9.19	8.88	7.45
Softness	8.88	8.44	8.69	9.50	8.56	8.00

**Table 5:** Subjective evaluation of the four-week evaluation.

Week	1	2	3	4	Mean
Days	4.3	3.5	3.6	3.3	3.7
Satisfaction	7.0	7.0	8.3	7.8	7.5
Ease of wear	8.7	7.8	9.0	8.8	8.6
Pain	1.9	2.5	1.9	2.1	2.1
Supportive	7.8	7.1	8.3	7.8	7.8
Comfort	8.5	7.8	8.8	8.5	8.4
Fit	8.0	8.3	9.2	8.1	8.4
Softness	8.7	8.1	8.3	8.7	8.5
Itchiness	3.8	4.0	3.6	2.5	3.5
Breathability	7.4	7.7	8.4	7.8	7.8
Range of motion	8.7	8.2	8.7	8.3	8.5

**Table 6:** Knitting parameter details.

Parameter			Value
Speed			0.7 m/s
Time			76 min
Gauge			15 L (12 needles)
Feeder no.			12
Needle Type			Sliding Needle
Density	1st row		4.8 mm
	2nd and 3rd rows		4.9 mm
	1 × 1 rib	Face	6.4 mm
		Back	5.4 mm
	Pointelle		5.7 mm
	Cup	Face	6.6 mm
		Spacer	6.0 mm
		Back	5.7 mm

**Table 7:** Summary of material performance.

Test Parameter	Standard	Device	Component	Result
Water vapor transmission rate (g/m^2^/day)	ASTM E96-22	Water Vapor Permeability Tester (SDL International Ltd., Stockport, Greater Manchester, UK)	Main fabric	712.40
			Bra Cups	556.20
			Under band	780.77
Thermal conductivity (W/m·K)	ASTM F1868	KES-F7 Thermo Labo II (Katō Tech Co., Ltd., Minami-ku, Kyoto, Japan)	Main fabric	0.05
			Bra Cups	0.06
			Under band	0.08
Air resistance (kPa·s/m)	ASTM D737	KES-F8 Air Permeability Tester (Katō Tech Co., Ltd., Minami-ku, Kyoto, Japan)	Main fabric	0.01
			Bra Cups	1.32
			Under band	0.21
Surface roughness (SMD)	JIS B0601	KES-FB4-A Surface Tester (Katō Tech Co., Ltd., Minami-ku, Kyoto, Japan)	Main fabric	7.33
			Bra Cups	0.65
			Under band	5.55
Young’s modulus (MPa)	EN 14704	Instron 4411 Tensile Tester (Instron, Norwood, MA, USA)	Main fabric	0.15
			Bra Cups	29.70
			Under band	0.82
Tensile strength (kPa)	ASTM D575	Instron 4411 Tensile Tester (Instron, Norwood, MA, USA)	Main fabric	0.25
			Bra Cups	194.29
			Under band	0.25

## Data Availability

The original contributions presented in this study are included in the article. Further inquiries can be directed to the corresponding author.
